# A Constellation of Rare Findings in a Case of Goldenhar Syndrome

**DOI:** 10.1155/2017/3529093

**Published:** 2017-05-21

**Authors:** Mitesh Bedi, Rakesh Kumar Jain, Vipin Kumar Barala, Abhimanyu Singh, Hiranmayi Jha

**Affiliations:** Department of Plastic Surgery, SMS Medical College, Jaipur, Rajasthan, India

## Abstract

An 18-month-old child presented with right macrostomia, bilateral preauricular skin tags, bilateral CTEV, squint in bilateral eyes, thoracic vertebral anomalies, right sided aortic arch, and associated left pulmonary agenesis. The patient did not have any associated respiratory symptoms. Ipsilateral pulmonary agenesis is considered as a rare association with Goldenhar syndrome and a case of contralateral pulmonary aplasia has been described as an even rarer association.

## 1. Introduction

Oculoauricular dysplasia, consisting of preauricular appendages, fistulas, and epibulbar dermoids, was first described in 1952 by the Swiss ophthalmologist Maurice Goldenhar and hence named Goldenhar syndrome. Gorlin et al. (1963) included vertebral anomalies as signs of the syndrome and suggested the name oculoauriculovertebral (OAV) dysplasia for this condition [1]. The incidence of Goldenhar syndrome has been reported to be 1 : 3500–1 : 5600 with a male to female ratio of 3 : 2 [2]. It is an incompletely understood spectrum of disorders of unknown etiology. It is a complex malformation of varying severity involving the structures arising from first and second branchial arches, first pharyngeal pouch, first branchial cleft, and primordia of the temporal bone.

## 2. Case Report

**Figure 1:**
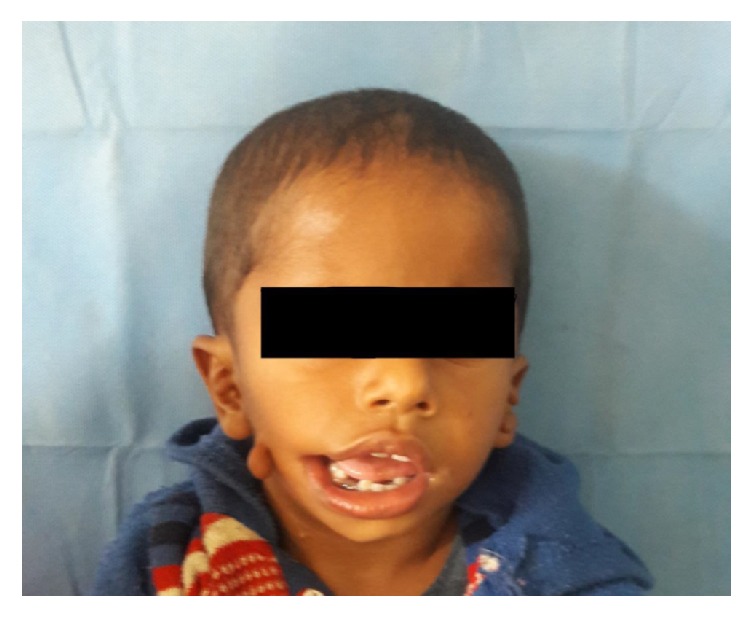
Frontal profile of patient.

**Figure 2:**
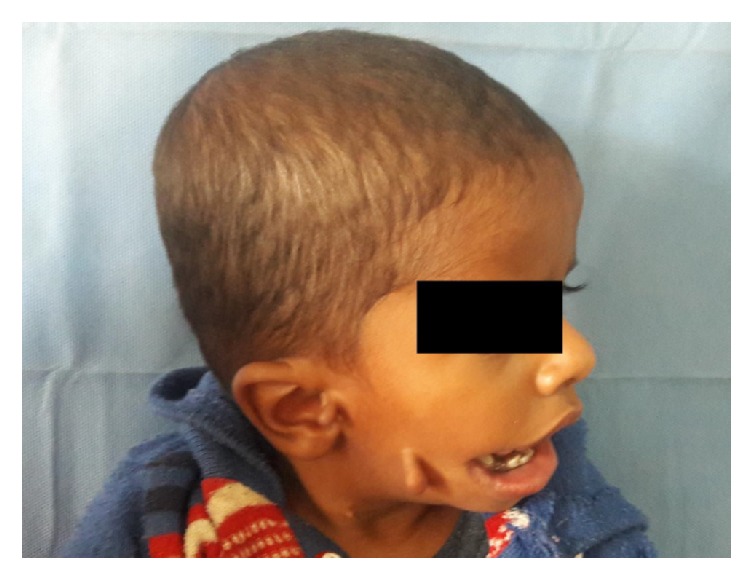
Side profile patient.

An 18-month-old child referred to the Department of Plastic and reconstructive surgery, SMS Medical College and Hospital, Jaipur, with complaints of macrostomia and bilateral preauricular skin tags. The child was born as a full term normal vaginal delivery with immediate cry and a birth weight of 1.75 kgs. There was no history of trauma to head and neck region or maternal exposure to teratogenic agents. The child was also noted for the presence of bilateral CTEV at birth. There were no signs of mental retardation or impairment of cognitive function. On examination, the head circumference was 43 cms. The child's weight was 13 kgs. And length was 74.93 cms. There were preauricular skin tags bilaterally along with macrostomia on right side (Figures 1 and 2) and squint in bilateral eyes; however, the child did not have any epibulbar dermoids or cyst.

The patient is undergoing treatment with serial casting for bilateral CTEV at present.

The patient has not had any previous history of respiratory problems.

**Figure 3:**
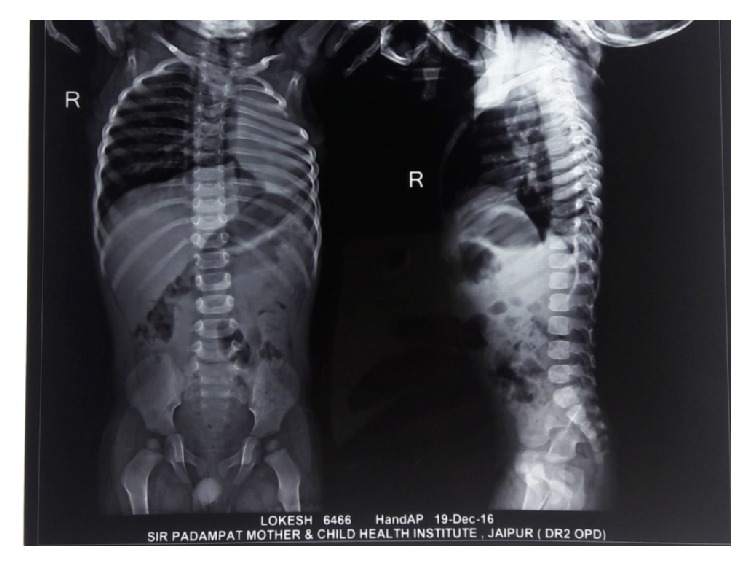
X-ray vertebrae.

**Figure 4:**
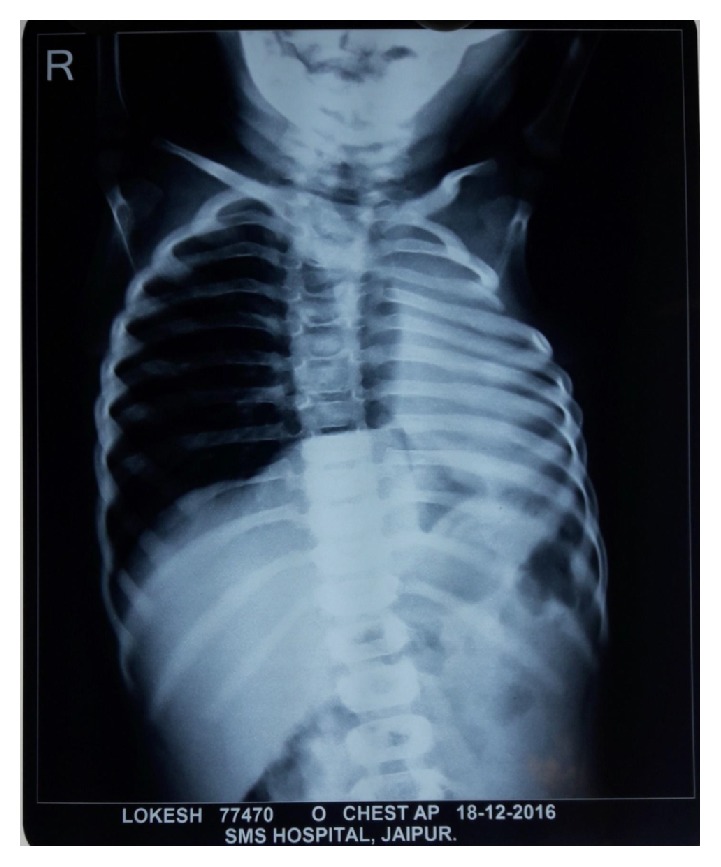
X-ray chest.

**Figure 5:**
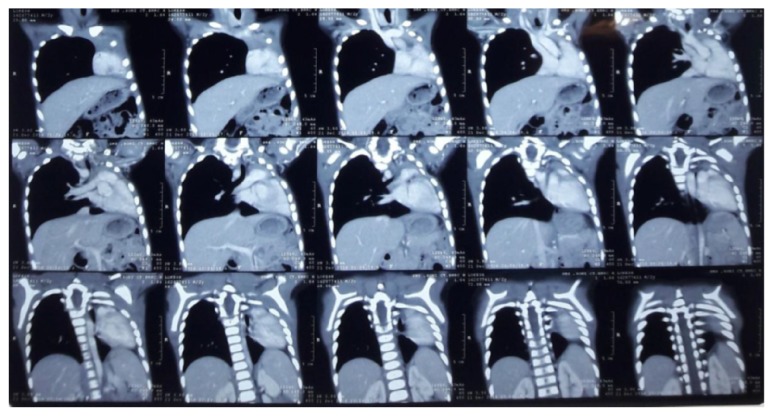
CT thorax.

Imaging studies include the following:

  X-ray hands: normal bone age
  X-ray vertebra (Figure 3): multiple thoracic vertebrae anomaly present including the following:
  - Mild scoliosis
  - Butterfly vertebrae (C5 and T1)
X-ray chest: complete loss of volume of left lung with contralateral hyperinflated lung and rib crowding in left hemithorax (Figure 4)
  USG abdomen: liver slightly enlarged and bright; kidneys: normal [3]
  Echocardiography: normal
  CT thorax (Figure 5):
  - Nonvisualization of left lung parenchyma and left bronchus with cardiac border shifted ipsilaterally
  - Right sided aortic arch

The child had a combination of rare findings including preauricular skin tags bilaterally along with macrostomia on right side, butterfly vertebrae (C5 and T1), left pulmonary agenesis, right sided aortic arch, and bilateral CTEV.

No genetic studies were carried out in the patient.

On the basis of these findings, a diagnosis of Goldenhar syndrome with pulmonary agenesis was made.

## 3. Management and Follow-Up

The patient was undergoing serial casting for bilateral club foot in orthopaedics department, at the time of presentation to plastic surgery department. The patient was operated on for correction of macrostomia and preauricular skin tags. The intraoperative as well as the postoperative course was uneventful.

## 4. Discussion

The classical features of Goldenhar syndrome patients involve ocular anomalies, including microphthalmia, anophthalmia, epibulbar dermoid (or lipodermoid) tumors, and eyelid colobomas, aural defects, such as preauricular tags, anotia, microtia, and hearing loss, vertebral abnormalities, such as scoliosis, hemivertebrae, and cervical fusion, and mandibular hypoplasia [4–6]. There may be an association with other major organ system anomalies including cardiac structural defects, renal agenesis, pulmonary agenesis, and vascular anomalies [7–9]. Our patient had no prior history of respiratory complaints, and the finding of pulmonary findings was an incidental finding on work-up of the patient. There have been only few reports of Goldenhar syndrome associated pulmonary anomalies in the literature [10, 11]. Usually, there is involvement of the lung on the same side as that of facial anomalies [10, 12, 13]. Contralateral pulmonary involvement is rarest association reported with Goldenhar syndrome [14, 15]. Although diagnosis is mainly clinical, radiographic investigations help to support the clinical diagnosis. Prenatal diagnosis is possible with considerable accuracy with ultrasound which may detect obvious defects. Since no specific genes have been linked to this syndrome, prenatal deoxyribonucleic acid testing cannot be used to diagnose the condition [16] Orthopaedic abnormalities in this syndrome usually include spinal anomalies but may also rarely involve congenital dislocation of the hip, Sprengel's deformity, clubfoot, and radial limb defects [17]. A combination of these rare findings in a single patient has been rarely reported in the literature to the best of our knowledge.
